# PKC-Theta in Regulatory and Effector T-cell Functions

**DOI:** 10.3389/fimmu.2015.00530

**Published:** 2015-10-13

**Authors:** Vedran Brezar, Wen Juan Tu, Nabila Seddiki

**Affiliations:** ^1^INSERM U955, Équipe 16 and Faculté de Médecine, Université Paris Est, Créteil, France; ^2^Vaccine Research Institute (VRI), Créteil, France; ^3^Faculty of Education, Science, Technology and Maths, University of Canberra, Canberra, ACT, Australia

**Keywords:** PKC-θ, immune synapse, regulatory T cells, effector T cells, immune interventions

## Abstract

One of the major goals in immunology research is to understand the regulatory mechanisms that underpin the rapid switch on/off of robust and efficient effector (Teffs) or regulatory (Tregs) T-cell responses. Understanding the molecular mechanisms underlying the regulation of such responses is critical for the development of effective therapies. T-cell activation involves the engagement of T-cell receptor and co-stimulatory signals, but the subsequent recruitment of serine/threonine-specific protein Kinase C-theta (PKC-θ) to the immunological synapse (IS) is instrumental for the formation of signaling complexes, which ultimately lead to a transcriptional network in T cells. Recent studies demonstrated that major differences between Teffs and Tregs occurred at the IS where its formation induces altered signaling pathways in Tregs. These pathways are characterized by reduced recruitment of PKC-θ, suggesting that PKC-θ inhibits Tregs suppressive function in a negative feedback loop. As the balance of Teffs and Tregs has been shown to be central in several diseases, it was not surprising that some studies revealed that PKC-θ plays a major role in the regulation of this balance. This review will examine recent knowledge on the role of PKC-θ in T-cell transcriptional responses and how this protein can impact on the function of both Tregs and Teffs.

## Introduction

Current global health challenges demand not only more effective and safer therapies to dampen undesired immune responses as in autoimmune diseases, inflammation, and transplant rejection, but also aim at boosting desired responses such as in cancer and infections. Hence, a major goal of immunology research has been to understand the regulatory mechanisms that underpin the rapid switch on/off of robust and efficient effector (Teffs) or regulatory (Tregs) T-cell responses. Thus, understanding the molecular mechanisms underlying the regulation of such responses is critical for the development of effective therapies.

Strong T-cell activation involves the engagement of T-cell receptor (TCR) and co-stimulatory signals. Subsequent recruitment of the serine/threonine-specific protein Kinase C-theta (PKC-θ) to the immunological synapse (IS) is instrumental for the formation of CARMA/BCL10/MALT (CBM) signaling complex in the cytoplasm (1–4). PKC-θ is the first PKC family member described to be recruited to the IS (5) and it plays an integral role in activating a range of signaling cascades that ultimately results in a transcriptional network in T cells. More recently, PKC-η was also described in immune synapse (6, 7) as well as PKC-ϵ (8).

Recent studies demonstrated that major differences between Teffs and Tregs occurred at the IS where its formation induces altered signaling pathways in Tregs, which are characterized by reduced recruitment of PKC-θ (9), suggesting that PKC-θ inhibits Tregs suppressive function in a negative feedback loop.

Expression level and stability of the transcription factor Foxp3 (forkhead box P3) are known to be critical for the development and function of *bona fide* Tregs. However, recent comprehensive analyses such as genome-wide and proteomics analysis revealed possible involvement of other molecular mechanisms in the development of Tregs. Fu and colleagues reported that combinations of Foxp3 with other transcription factors are able to induce a common Treg-type gene expression pattern, which cannot be achieved solely by Foxp3 (10). Molecules such as Smad3, NFAT, and AP-1 have been identified to initiate and/or enhance Foxp3 transcription. While some gene expression in Tregs is directly modulated by the binding of Foxp3 to their promoters or enhancers, other gene expression requires interaction of Foxp3 with other transcription factors. It remains to determine whether PKC-θ can directly modulate Foxp3 transcription to then inhibit Tregs suppressive activity or requires implication of other transcription factors.

Signaling kinases have emerged as a new class of chromatin-associated enzymes that act as an intermediary between cytoplasmic and chromatin modifications. This is exemplified by Hog1 in yeast or the human homolog, p38α, which activates target gene expression during mitotic stress by interacting with ATP-dependent chromatin remodelers and other kinases, e.g., MSK1/2 to phosphorylate H3 Ser10 and 28 (11). Due to their nuclear-localizing signal (NLS) (12), PKC family members represent a novel class of chromatin-associated kinases that alternate between the cytoplasm and nucleus (13–16). Their role in T-cell transcriptional responses needs to be unequivocally proven. Therefore, further investigations are needed.

## PKC-θ and T-Cell Responses

### PKC-θ Structure and Function

PKC includes a large family of homologous serine/threonine protein kinases that is widely conserved in eukaryotes. In mammals, there are 12 isoforms that are identified and subdivided into three groups based on their divergent regulatory domains and their second messenger requirements for activation: the conventional PKCs (cPKCs: α, βI, βII, and γ); novel PKCs (nPKCs: δ, ϵ, η, and θ); and atypical PKCs (aPKCs: ι/λ) and ζ) (17).

**Figure 1:**
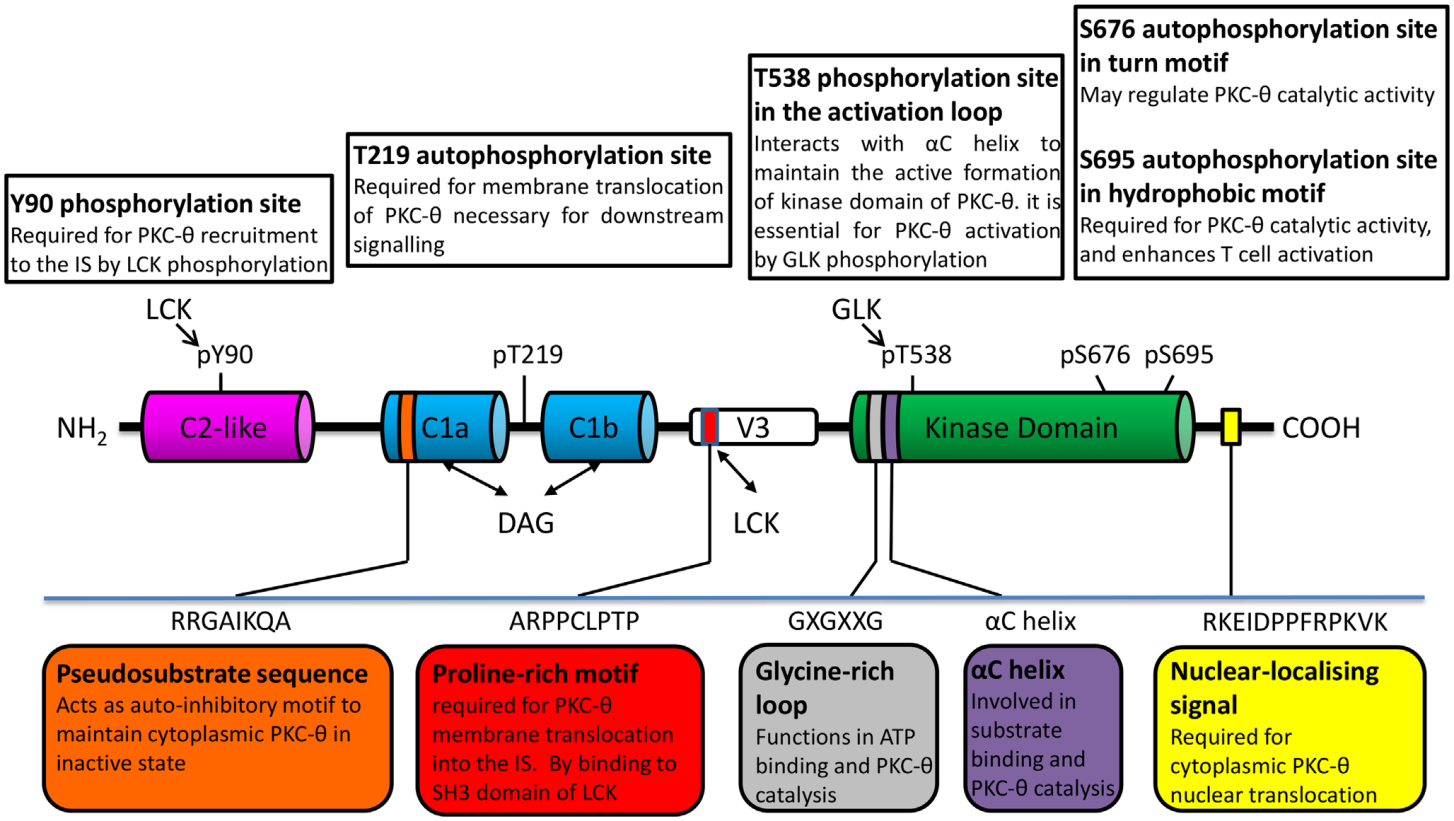
**PKC-**θ**/PKC family structure important to its catalytic modulation and cellular translocation**.

Similar to other PKC isoforms, the basic structure of PKC-θ is composed of an N-terminal regulatory domain and a highly homologous conserved C-terminal kinase domain, which is tethered by unique V3 hinge region. Recently, the polyproline motif in the V3 hinge region has shown an essential role for PKC-θ translocation into the IS (18). The regulatory region of PKC-θ contains N-terminal C2-like domain which is Ca^2+^ irresponsive, followed by two tandem cysteine-rich zinc finger C1 domains (C1a and C1b) that are responsible for the binding to the second messenger diacylglycerol (DAG) or phorbol ester (such as phorbol-12-myristate-13-acetate, PMA) (Figure 1). Due to the structural difference of C2 domain, PKC-θ is activated by DAG/PMA but in Ca^2+^ independent manner, compared to cPKCs that require both DAG/PMA and Ca^2+^ (19).

The intrinsic PKC-θ kinase activity is regulated through an allosteric mechanism, which leads to the change in PKC-θ conformation between “closed/inactive” and “open/active” state (20, 21). Upon the initial receptor stimulation, PKC-θ is recruited to plasma membrane via membrane-resident DAG binding to its C1 domain. This triggers the conformational change of PKC-θ from “close” to “open” state, which allows its activation loop in the kinase domain to be accessible for the phosphorylation by germinal center kinase-like kinase (GLK, also known as MAP4K3) (22). There are several phosphorylation sites that have been identified in PKC-θ. These sites play distinct roles in its kinase activity and membrane translocation. Amongst them, Thr-538 in the activation loop, which is critical for kinase activation (23). Other phosphorylation sites include Ser-676 (in turn motif of kinase domain), Ser-695 (in hydrophobic motif of kinase domain), and PKC-θ unique sites (Tyr-90 in C2-like domain and Thr-219 in C1 domain) (24) (Figure 1). Upon phosphorylation, pseudosubstrate is unlocked from kinase domain, allowing subsequent catalytic activation of PKC-θ and its downstream signaling functions required for T-cell survival, proliferation, and homeostasis (25). However, the direct kinases and/or specific regulator for these phosphorylation sites on PKC-θ are still poorly understood.

### PKC-θ and T-Cell Responses: Importance of Immune Synapse Formation

Despite the fact that different PKC isoforms, including PKC-α, δ, ϵ, η, θ, and ζ are expressed at various levels in T cells (26, 27), PKC-θ is the most studied protein kinase. Its presence in the IS following antigen stimulation of T cells is well determined (28). However, besides PKC-θ, PKC-η, and PKC-ϵ are both present in IS (6–8). The role of PKC-η is getting more attention in the recent years as reviewed recently elsewhere (29).

A digital three-dimensional imaging analysis during T cell and antigen-presenting cells (APCs) interactions revealed a bull’s eye structure of the IS. Three distinct subregions were identified: central supramolecular activation cluster (cSMAC), peripheral SMAC (pSMAC), and distal SMAC (dSMAC) (30, 31). An intermediate ring of pSMAC surrounds the central core cSMAC, which is enriched with cognate integrin lymphocyte function-associated antigen 1 (LFA-1, also known as α4β7 integrin) and intercellular adhesion molecule 1 (ICAM-1) (32).

**Figure 2:**
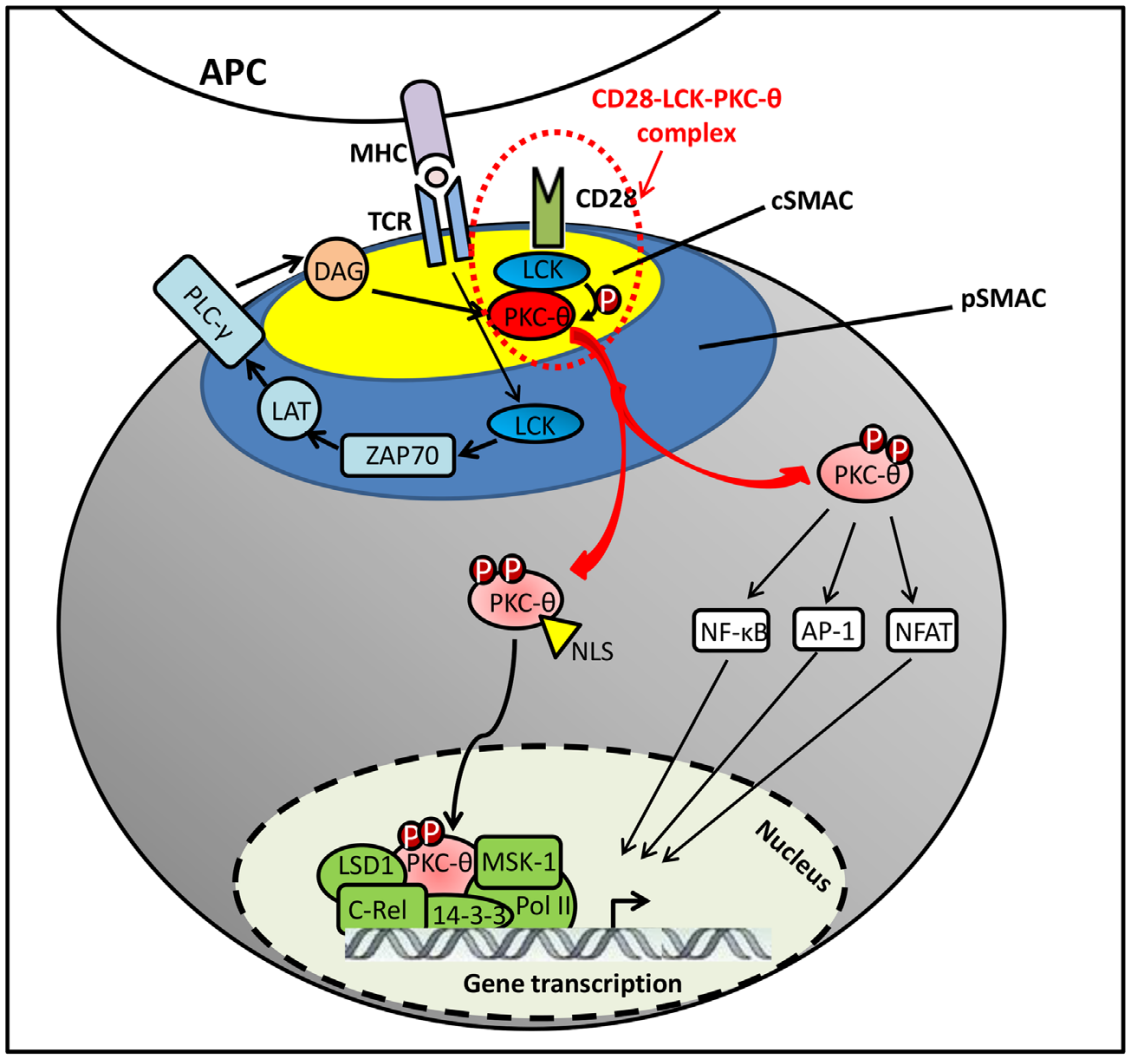
**PKC-**θ** translocation in immune synapse**.

The initial signaling from TCR/CD28 stimulation was found to induce the DAG accumulation on plasma membrane, which recruits PKC-θ to the IS by binding to its C1 domains (24). However, DAG–C1 domain interaction itself does not seem sufficient for the selective translocation of PKC-θ to the cSMAC. Other coeffector engagement is required for the selective PKC-θ recruitment to cSMAC (Figure 2). Particularly, Lck tyrosine kinase plays an essential dual role in regulating CD28–PKC-θ complex formation and PKC-θ conformational change in this IS translocation. First, Lck serves as a linker/adaptor in CD28–PKC-θ complex. Also, Src-homology (SH)2 or SH3 domains in Lck is associated with a phosphotyrosine (pTyr)-containing C-terminal proline (Pro)-rich motif (Pro–pTyr^188^–Ala–Pro) in mature CD28 (1, 33). More recently, Kong et al. have also shown that Pro-rich motif within the V3 domain binds to the SH3 domain of Lck (18). Therefore, the most likely binding model was suggested as a PKC-θ/Lck/CD28 trimolecular signaling complex, in which the SH2 domain of Lck interacts with phosphorylated Tyr-188 of the CD28 cytoplasmic tail and the SH3 domain of Lck binds to Pro-rich motif in the V3 domain of PKC-θ (32). Moreover, when PKC-θ V3 pro-rich motif is mutated, it disrupts the CD28–PKC-θ complex formation and impairs PKC-θ-mediated downstream T-cell activation and differentiation in Th2 and Th17 cells (18). In addition, Lck has also shown the ability of directly phosphorylating PKC-θ at Tyr-90 in its C2-like domain both *in vitro* and *in vivo* (1). However, there is no direct evidence to show that Tyr-90 phosphorylation participates in PKC-θ membrane translocation, and it still remains unclear how Tyr-90 phosphorylation regulates the PKC-θ conformational alteration and kinase activation. Along with DAG binding and Tyr-90 phosphorylation initiated active conformation of PKC-θ, Thr-538 is directly phosphorylated by GLK in the activation loop that is responsible for the stability of PKC-θ active conformation. Although Thr-538 may not directly regulate the IS recruitment of PKC-θ, it enables the accessible kinase domains to undergo autophosphorylation at other phosphorylation site, such as Thr-219, which is required for proper translocation of PKC-θ to the cSMAC (24, 34).

In contrast to the long-lived symmetric IS formation that is required for productive T-cell activation and differentiation, transient asymmetric synapse is able to induce T-cell anergy in response to weak signal input, such as in the absence of CD28 co-stimulatory signal (35). The conversion between IS and kinapses was found to be regulated by PKC-θ and Wiscott Aldrich Syndrome protein (WASp). PKC-θ negatively controls the stability of IS, while WASp restores the IS in the absence of PKC-θ activity (36).

It is well documented that PKC-θ plays a critical role in T-cell activation, proliferation, and differentiation. *Ex vivo* studies have shown that PKC-θ is involved in the activation of NF-κB, activation protein-1 (AP1), and nuclear factor of T cells (NFAT) (37–41). In resting T cells, NF-κB is sequestered in the cytoplasm by IkB that binds to its NLS. Upon the TCR/CD28 activation, PKC-θ phosphorylates membrane-associated guanylate kinase (MAGUK) domain-containing protein 1 (CARMA1) on its serine residues, resulting in the recruitment of B-cell lymphoma/leukemia 10 (BCL10) and mucosa-associated lymphoid tissue 1 (MALT1) to form an active CARMA1–BCL10–MALT1 signaling complex. Then it promotes the activation of IKK complex to phosphorylate the inhibitory IkB for its degradation, leading to NF-κB nuclear translocation for transcriptional programs required for T-cell activation (38). Recently, PKC-θ has been identified as an essential component in OX40 signalosome, containing molecules such as OX40, TRAF2, RIP2, IKKα/β/γ, as well as the CBM complex. This process has been shown to be independent of TCR engagement (42).

Although the importance of PKC-θ catalytic activity is extensively addressed in T cells, the chromatin-associated role of this signal transduction kinase is still poorly understood. Upon T-cell activation, PKC-θ is translocated to the nucleus via NLS (12), forming an active chromatin-anchored complex that includes RNA polymerase II, the histone kinase MSK-1, lysine specific demethylase 1 (LSD1), and the adaptor molecule, 14-3-3ζ. This complex then localizes to the proximal promoter and coding regions of inducible immune responsive genes in human T cells (16). Moreover, the formation of this nuclear PKC-θ-containing transcriptional complex at regulatory regions of gene targets is persistent, which contrasts to its rapid association with signaling molecules at the IS. In addition, a chromatin immunoprecipitation (ChIP)-on-ChIP assay showed that PKC-θ also negatively regulates a distinct cluster of microRNA transcription by tethering at their promoter regions (16). More recent studies indicated that chromatin-associated NF-κB is required for the assembly of PKC-θ-containing active transcription complex. Moreover, NF-κB negatively regulates miR-200 transcription by forming a repressive complex on target gene impeding the formation of PKC-θ active transcription complex (43). However, further studies are required to determine the functional differences between cytoplasmic and nuclear-targeted PKC-θ regulation and their contribution to transcriptional regulations in T cells.

The role of PKC-θ is evidently more diverse since *in vivo* studies on *Prkcq*^−/−^ mice in different disease models showed differential requirements by distinct T-cell subsets. The studies have shown that PKC-θ is dispensable for the differentiation and effector function of Th1 cells (44, 45). PKC-θ-deficient mice showed intact CTL responses against intracellular bacterium Leishmania major (44), LCMV (46), and murine gamma-herpesvirus 68 infection (47). However, Th2 cell proliferation and differentiation is significantly defective in *Prkcq*^−/−^ mice, which may reflect its important role in upregulating the GATA-3 expression (48). PKC-θ was also found to be essential for the induction of effective Th2 response against allergens or helminth infection (44). More recent studies showed that PKC-θ was required in the induction of graft-versus-host disease (GvHD) and alloreactive T-cell mediated immune response. However, it was dispensable for inducing graft-versus-leukemia (GvL) response in bone marrow transplantation (BMT) mice model (49). In sharp contrast to the positive role of PKC-θ in the promotion of Th2 effective immune response, recent studies indicated that PKC-θ negatively regulates the Tregs cells suppressive function.

## PKC-θ and Tregs

Regulatory T cells play a pivotal role in immune homeostasis. This CD4^+^ T-cell subset is important in autoimmunity prevention but also for avoiding any exaggerated immune responses that would be harmful to the host (50). Tregs could also be deleterious as is the case in cancer where the suppression of effector T cells (Teffs) responses might lead to tumor growth (51). In case of HIV infection, their role is ambiguous. In this context, the suppression of immune activation is considered beneficial while the suppression of HIV-specific responses, deleterious (52). Tregs develop in the thymus but some are induced in the periphery following differentiation of naïve CD4^+^ T cells after acquiring appropriate signals. The expression of transcription factor FoxP3 as well as several other molecules such as CTLA-4, CD39, and CD25 (IL-2Rα) are associated with Tregs function (53). Interleukin 2 (IL-2) is crucial for their expansion, survival as well as function (54). As is the case for Teffs, Tregs also need TCR stimulation to exert their function through contact-depending mechanisms (55). However, the signaling pathways in Tregs are slightly different from Teffs. A recent study which analyzed kinase-based signaling networks in Tregs and Teffs reported that only 11 out of 185 kinases are differently expressed between these two subsets (56). Although it is thought that PKC-θ plays an important role in Tregs, its function has not been extensively studied. By far, the most comprehensive analysis of its role comes from Zanin-Zhorov et al. (9). To study signaling in ISs of Tregs, these authors developed a system where Tregs and Teffs were added to a planar bilayers containing ICAM-1 and aCD3 antibodies. Tregs formed stronger and more stable synapses than Teffs (9). They then studied PKC-θ as it mediates the breaking of ISs (36) and found significantly lower levels of PKC-θ recruited in the synapses of Tregs, in comparison with Teffs (9). Moreover, PKC-θ was found sequestered at the distal pole in Tregs, away from TCR suggesting that different localization of PKC-θ drives different functions. These authors also found that pretreatment with PKC-θ inhibitor enhanced Tregs function as they became more suppressive in comparison with non-treated Tregs (9). This finding is in discrepancy with earlier observations from Gupta et al. (57) who used a knock-out mice model to study the role of PKC-θ in Tregs function. This study revealed that Tregs from PKC-θ-deficient mice were equally suppressive to Tregs from wt mice (57). However, they showed that PKC-θ is important in Tregs development as they were significantly reduced in PKC-θ-deficient mice. This deficiency is probably due to the enhancing effect PKC-θ has on FoxP3 expression, through calcineurin/NFAT pathway (57). The other possibility is the reduced IL-2 production by Teffs in PKC-θ-deficient mice that impacts Tregs survival, proliferation, and function (54). These discrepancies between the two studies are most probably due to major differences in the model/system used. Also, targeted inhibition, either by PKC inhibitor or siRNA, which was used by Zanin-Zhorov and colleagues, might have not been specific only for PKC-θ but influenced other pathways that could have impacted Tregs’ function (58).

**Figure 3:**
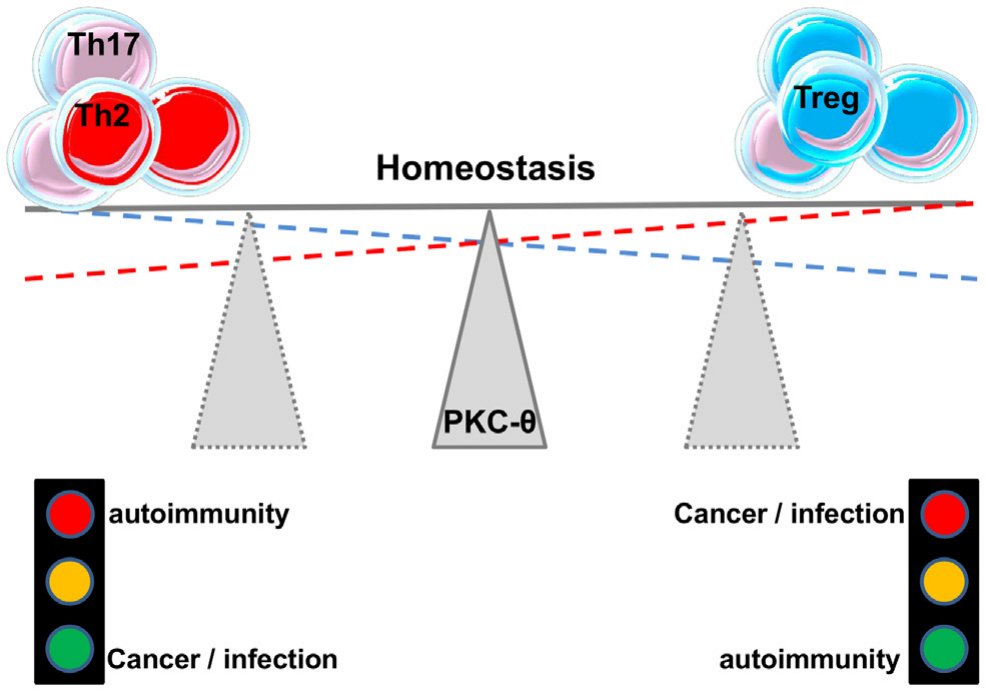
**PKC-**θ** and the balance between effector and regulatory responses**.

As mentioned before, Tregs can be of thymic origin but can also be induced in the periphery (iTregs). Ma et al. (59) studied the role of PKC-θ in the development of iTregs and found that PKC-θ-mediated signals through Akt-Foxo1/3A pathway inhibit the differentiation of iTregs from naïve CD4^+^ T cells in the presence of TGF-β. Blocking PKC-θ either by an inhibitor or by gene knockdown reversed the inhibition of iTregs differentiation. Altogether these studies suggest an important role for PKC-θ in fine-tuning the balance between Teffs and Tregs responses (Figure 3). This makes this protein kinase an attractive drug target, a feature that will be further discussed in another chapter below. In addition, the association between kinase PKC-η with CTLA-4 and its recruitment to the Tregs’ IS has recently been described (60). Defective activation of this complex in PKC-η-deficient Tregs cells was associated with reduced depletion of CD86 from APCs by Tregs. These results reveal a CTLA-4-PKC-η signaling axis required for contact-dependent suppression and implicate this pathway in the regulation of the balance between regulatory and effector mechanisms in different diseases.

## Role of PKC-θ in Human Diseases

As discussed above, PKC-θ impacts on the function of both Tregs and Teffs. As the balance of these subsets has been shown to be central in several diseases, it was not surprising to find that PKC-θ plays a major role in these processes.

### Autoimmunity

Autoimmunity often results from an aberrant immune response following the activation of self-reactive T cells. Very valuable information about the implication of PKC-θ in autoimmune diseases came from PKC-θ-deficient mice. It was shown in several studies that PKC-θ-deficient mice were resistant to experimental autoimmune encephalomyelitis after injection of myelin oligodendrocyte glycoprotein (MOG) (61–63). These mice presented less T-cell infiltration as well as diminished production of proinflammatory cytokines IFN-γ, TNF, and IL-17 following the immunization. Similar role was observed in other autoimmune syndromes such as collagen-induced arthritis (64), colitis (9, 62), and myosin-induced autoimmune myocarditis (65). Despite the discrepancies reported from the studies on the role of PKC-θ in Tregs suppressive function (9, 57), it has been reported that in the colitis model, the blockage of Tregs’ PKC-θ is highly protective (9). Interestingly, when Teffs were treated with the same inhibitor before transfer, mice were not protected from colitis indicating the preferential role for Tregs-mediated suppression of the disease through PKC-θ pathway. These findings generated large interest to study PKC-θ in human autoimmune diseases. Genome-wide association studies (GWAS) identified specific single nucleotide polymorphisms (SNP) within Prkcq locus associated with type 1 diabetes (T1D), rheumatoid arthritis (RA), and celiac disease (66–69). Recent findings showed an important impairment of Tregs in RA patients (70). Zhanin-Zhorov et al. used samples from patients with various diseases’ severity and isolated Tregs. They showed that inhibition of PKC-θ increased the suppressive activity of these cells (9). Moreover, they found that PKC-θ inhibition renders Tregs resistant to inhibition by TNF-α that is known to inhibit Tregs activity by down-regulating FoxP3 (70).

### Cancer

On the other side of the spectrum from the autoimmune diseases, at least from immune standpoint, is cancer. Whereas in autoimmune settings Tregs play a major beneficial role, in cancer settings, they are considered to be deleterious as they are shown to suppress anti-tumor responses (71). Can fine-tuning of PKC-θ expression and its positioning in immune synapse be a potential target in cancer drug trials is still under question.

Several studies describe the role of PKC-θ in cancer settings. In gastrointestinal stromal tumors (GISTs) and Ewing’s sarcoma PKC-θ could be used as a specific marker of the disease (72–75). These studies suggest that PKC-θ overexpression facilitates the diagnostics of tumors even in the case where traditional markers are absent. However, the role in the pathogenesis is still unclear.

The implication of many other PKCs in cancer is also identified and several clinical trials are already ongoing (76). Whether there is a role for PKC-θ pathway in antitumoral T-cell response and what are the implications for drug development is still to be answered. The fact that PKC-θ seems to be expressed also by the tumor cells invites caution.

### HIV

T-cell activation is a crucial step in HIV infection and replication. Synthesis of dNTPs, increased ATP levels, and activation of transcription factors such as NF-κB, NF-AT, and AP-1 are all shown to be necessary for HIV replication (77). Therefore, being essential for several T-cell activation pathways, PKC-θ could play a major role in HIV infection.

Very early studies showed that Nef interacts preferentially with PKC-θ. This interaction is independent of calcium and enhanced by phospholipid activators of PKC. More importantly, the authors found a net loss of PKC-θ in Nef-expressing cells following stimulation. The conclusion was that this phenomenon may contribute to the various impairments of T-cell function associated with HIV infection (78).

More recent studies have shown that HIV infection employs PKC-θ to enhance HIV-1 replication. In turn, it also upregulates PKC-θ phosphorylation in its activation loop as a positive feedback in CD4^+^ T cell. Moreover, HIV-1 replication was reduced in CD4^+^ T cells in the absence of PKC-θ activity, either by using pharmacological inhibitor, rottlerin, or employing RNA interference (RNAi) strategy (79).

However, how PKC-θ affects T-cell responses against the virus itself was not studied. Some suggest that PKC-θ is an interesting target for decreasing immune activation in HIV-infected patients and that this goal could be achieved without major immunosuppression (80).

## PKC-θ and Targeting for Immune Interventions

Impairing the PKC-θ activity is believed to be a promising therapeutic strategy against the undesired immune response, such as Th2-mediated allergies, Th17-associated autoimmune diseases (63), and GvHD (49), meanwhile preserving the beneficial Th1 and CTL anti-pathogen immunity (47, 81) as well as GvL response in BMT (49). Therefore, pharmaceutical companies have dedicated considerable efforts to develop PKC-θ inhibitors.

The main PKC-θ inhibitors were designed as ATP competitors to block PKC-θ kinase activity. So far, the most successful is sotrastaurin (AEB071) (see Table 1), which has reached substantial progress in phase I clinical trials in psoriasis and phase II clinical trial in renal transplantation (82–84). AEB071 is a “multikinase” inhibitor with strong specificity for PKC-θ, PKC-α, and PKC-β at low picomolar concentration and less preference for other nPKCs of PKC-δ, PKC-ϵ, and PKC-η at nanomolar concentration (84). Consistent with previous findings of selective regulation of T-cell development in PKC-θ^−^/^−^ animal model, AEB071 inhibited TCR/CD28-mediated T-cell proliferation, GvHD and allograft rejection (85–87), but retained T-cell antiviral response (83). Although both PKC-θ and PKC-α are inhibited following AEB071 treatment, NFAT activation seems not impaired (88).

**Table 1:** **PKC-**θ** inhibitor in human diseases and clinical trials**.

Mechanism of inhibition	Potential drug	Target	Disease treatment	Clinical trial
ATP competitor	Sotrastaurin (AEB071)	cPKCs: PKC-α and PKC-β and nPKCs: PKC-θ, PKC-δ, PKC-ϵ, PKC-η	Psoriasis, renal transplantation, uveal melanoma, and large B-cell lymphoma.	phase I and phase II
	R524	PKC-θ and PKC-α	GvHD	N/A
	Enzastaurin (Ly317615)	PKC-θ and PKC-β	Multiple myeloma, large B-cell lymphoma, maybe GvHD	phase II
	Compound C20 (C20)	PKC-θ	Rheumatoid arthritis (RA)	N/A
	Compound C27 (C27)	PKC-θ	N/A	N/A
Phosphorylation inhibitor	4-hydroxy-3-methoxycinnamaldehyde (4H3MC)	PKC-α, PKC-θ, and PKCs/λ	Maybe in T cell-mediated immune diseases	N/A
	CGX1079 and CGX0471	PKC-θ’s T538 phosphorylation	HIV infection	N/A
Nuclear translocation inhibitor	N/A	PKC-θ	N/A	N/A

Several other attractive PKC-θ inhibitors can be valuable in the treatment of autoimmune diseases. PKC-θ specific inhibitory compound C20 has been reported to increase the suppressive function of Tregs cells from RA patients (9). In another study, PKC inhibitor R524 was designed to inhibit both PKC-θ and PKC-α catalytic activity at the nanomolar concentration. Haarberg and colleagues (89) showed that R524 impaired CD4^+^ T-cell proliferation and cytokine production, and significantly attenuated GvHD symptoms in myeloablative preclinical mouse models of allogeneic hematopoietic cell transplantation (HCT). Another potential PKC inhibitor is enzastaurin (Ly317615), which is orally bioavailable ATP inhibitor originally identified as PKC-β inhibitor (90). This study has shown that Ly317615 has long-term activity on anti-proliferation and pro-apoptosis in both solid and hematologic cancer. Currently, Ly317615 has been evaluated in different clinical trials, including phase II trials in multiple myeloma (91) and diffuse large B-cell lymphoma (92). However, *in vitro* Upstate kinase profiler data showed that Ly317615 inhibits PKC-θ fivefold more potently than PKC-β at 1 μmol/L concentration (90). Therefore, Ly317615 may act as PKC-θ inhibitor to prevent GvHD while retaining GvL response (93). It is suggested that highly specific inhibition of PKC-θ will promote a better efficacy and safety in the treatment of autoimmune diseases without causing overt immunosuppression (94). However, since PKC-θ and PKC-δ share highly conserved ATP-active region with only a single residue difference (Tyr108 in PKC-θ and Phe108 in PKCδ), it has been a challenging task to develop a small molecule compound with a high degree of specificity toward PKC-θ. In 2013, Jimenez et al. designed a novel compound 27 (C27), with an excellent selectivity toward PKC-θ comparing to other PKC isoforms and non-kinase targets. Moreover, it did not show cross-reactivity against other proximal TCR kinases. C27 inhibitor showed encouraging success at the preclinical level to effectively inhibit IL-2 production in a mouse model of staphylococcal enterotoxin B-induced IL-2 release (SEB IL-2 model) (95), which makes it a potent and specific PKC-θ inhibitor candidate for therapy in autoimmune diseases.

In addition to the PKC-θ inhibitors that act as ATP competitors, other inhibitors that negatively regulate PKC-θ phosphorylation have been developed and studied in several disease models. 4-hydroxy-3-methoxycinnamaldehyde (4H3MC) was identified as a potential PKC isotypes inhibitor, preferentially inhibiting PKC-α, PKC-θ, and PKCsι. 4H3MC ablates PKC-θ phosphorylation and impairs its translocation to the IS, subsequently inhibiting IL-2 production in Jurkat T cells and human leukocytes. In addition, it also blocks the phosphorylation of ERK and p38, which results in impairing the activation of AP-1, NFAT, and NF-κB (96). This suggests that it may be an attractive PKC inhibitor candidate against T-cell-mediated autoimmune diseases. Two PKC-θ-specific inhibitors – CGX1079 and CGX0471 – were investigated as potential therapeutic adjuvants for the antiretroviral therapy (ART) in HIV infection (80). These two inhibitors impair PKC-θ kinase activity by blocking PKC-θ phosphorylation at T538 and prevent its translocation to the IS, which subsequently impairs the activation of NF-κB, AP-1, and NFAT and decreases viral transcription. Moreover, CGX1079 and CGX0471 reduce HIV-1 retrotranscription by inhibiting SAMHD1 phosphorylation at T592 that associates with attenuated proviral integration in PBMCs isolated from HIV-infected patients with ART treatment (Table 1). Despite CGX1079 and CGX0471 retardation of T-cell proliferation, these compounds did not completely compromised T-cell function, particularly CD8 anti-viral activity, avoiding general immunosuppression. Altogether, CGX1079 and CGX0471 are promising PKC-θ inhibitors that can reduce reservoir size and preserve CTL function against HIV-1 infection (80).

However, there are still some challenges that need to be taken into consideration for developing the full therapeutic potential of PKC-θ specific inhibitors in clinical applications. First, the double-edged sword feature of PKC-θ regulation in Teffs and Tregs. Tregs cell function enhancement may be favored in autoimmune diseases, while impaired Teffs cell function is not desired in tumor-specific T-cell responses. Second, as the kinase domain is well conserved among all PKC isoforms, even crossover other protein kinase members, using ATP competitors to specifically target PKC-θ would be difficult. Therefore, allosteric kinase inhibitors emerge as more specific and less toxic therapeutic candidates in the context of human diseases. As allosteric kinase inhibitors target more divergent regulatory regions and regulate the conformational changes required for kinase activation, rather than highly conserved catalytic regions of PKC-θ, their use would be more suitable.

Moreover, the pro-rich motif in the V3 hinge domain of PKC-θ that was recently identified by Kong et al. may serve as an attractive target for allosteric inhibition. This study indicated that the V3 hinge region is sufficient to trigger PKC-θ translocation into the IS or cSMAC. Importantly, this pro-rich motif in the V3 hinge domain is unique to PKC-θ and promises a high specificity in kinase activity inhibition (18). Taken together, those less conserved and much more flexible hinge regions may become potential targets for optimal PKC inhibitor design.

### Perspectives

The chromatin-tethered role of PKC-θ has been recently identified to be essential in the regulation of inducible gene transcription in human T cells by forming an active chromatin-anchored complex that associates with RNA polymerase II, the histone kinase MSK-1, LSD1, and the adaptor molecule, 14-3-3ζ (16). In addition, blocking PKC-θ nuclear translocation impairs T-cell activation and inducible gene expression by targeting its C-terminal NLS motif, which suggests the importance of its nuclear role in T-cell activation (Li et al., unpublished data). Therefore, it may provide an alternative approach to inhibit PKC-dependent T-cell function by inhibiting its nuclear role besides the catalytic activity. However, further studies are required because very little is known about the molecular mechanism of nuclear-tethered PKC-θ in regulation of T-cell immune responses.

## Conflict of Interest Statement

The authors declare that the research was conducted in the absence of any commercial or financial relationships that could be construed as a potential conflict of interest.

## References

[1] Liu Y Witte S Liu YC Doyle M Elly C Altman A. Regulation of protein kinase Cθ function during T cell activation by Lck-mediated tyrosine phosphorylation. J Biol Chem (2000) 275(5):3603–9. 10.1074/jbc.275.5.3603 10652356

[2] Lin X Wang D. The roles of CARMA1, Bcl10, and MALT1 in antigen receptor signaling. Semin Immunol (2004) 16(6):429–35. 10.1016/j.smim.2004.08.022 15541657

[3] Thome M CARMA1, BCL-10 and MALT1 in lymphocyte development and activation. Nat Rev Immunol (2004) 4(5):348–59. 10.1038/nri1352 15122200

[4] Weil R Israël A. T-cell-receptor- and B-cell-receptor-mediated activation of NF-kappaB in lymphocytes. Curr Opin Immunol (2004) 16(3):374–81. 10.1016/j.coi.2004.03.003 15134788

[5] Arendt CW Albrecht B Soos TJ Littman DR. Protein kinase C-theta: signaling from the center of the T-cell synapse. Curr Opin Immunol (2002) 14(3):323–30. 10.1016/S0952-7915(02)00346-1 11973130

[6] Singleton KL Roybal KT Sun Y Fu G Gascoigne NR van Oers NS Spatiotemporal patterning during T cell activation is highly diverse. Sci Signal (2009) 2(65):ra15. 10.1126/scisignal.2000199 19351954 PMC2694444

[7] Fu G Hu J Niederberger-Magnenat N Rybakin V Casas J Yachi PP Protein kinase C η is required for T cell activation and homeostatic proliferation. Sci Signal (2011) 4(202):ra84. 10.1126/scisignal.2002058 22155788 PMC3242502

[8] Quann EJ Liu X Altan-Bonnet G Huse M. A cascade of protein kinase C isozymes promotes cytoskeletal polarization in T cells. Nat Immunol (2011) 12(7):647–54. 10.1038/ni.2033 21602810 PMC3119370

[9] Zanin-Zhorov A Ding Y Kumari S Attur M Hippen KL Brown M Protein kinase C-θ mediates negative feedback on regulatory T cell function. Science (2010) 328(5976):372–6. 10.1126/science.1186068 20339032 PMC2905626

[10] Fu W Ergun A Lu T Hill JA Haxhinasto S Fassett MS A multiply redundant genetic switch ‘locks in’ the transcriptional signature of regulatory T cells. Nat Immunol (2012) 13(10):972–80. 10.1038/ni.2420 22961053 PMC3698954

[11] Pokholok DK Zeitlinger J Hannett NM Reynolds DB Young RA. Activated signal transduction kinases frequently occupy target genes. Science (2006) 313(5786):533–6. 10.1126/science.1127677 16873666

[12] DeVries TA Neville MC Reyland ME. Nuclear import of PKCδ is required for apoptosis: identification of a novel nuclear import sequence. EMBO J (2002) 21(22):6050–60. 10.1093/emboj/cdf606 12426377 PMC137198

[13] Martelli AM Faenza I Billi AM Falà F Cocco L Manzoli L. Nuclear protein kinase C isoforms: key players in multiple cell functions? Histol Histopathol (2003) 18(4):1301–12. 12973696 10.14670/HH-18.1301

[14] Hurd PJ Bannister AJ Halls K Dawson MA Vermeulen M Olsen JV Phosphorylation of histone H3 Thr-45 is linked to apoptosis. J Biol Chem (2009) 284(24):16575–83. 10.1074/jbc.M109.005421 19363025 PMC2713519

[15] Metzger E Imhof A Patel D Kahl P Hoffmeyer K Friedrichs N Phosphorylation of histone H3T6 by PKCbeta(I) controls demethylation at histone H3K4. Nature (2010) 464(7289):792–6. 10.1038/nature08839 20228790

[16] Sutcliffe EL Bunting KL He YQ Li J Phetsouphanh C Seddiki N Chromatin-associated protein kinase C-θ regulates an inducible gene expression program and MicroRNAs in human T lymphocytes. Mol Cell (2011) 41(6):704–19. 10.1016/j.molcel.2011.02.030 21419345

[17] Mellor H Parker PJ The extended protein kinase C superfamily. Biochem J (1998) 332(Pt 2):281–92. 10.1042/bj3320281 9601053 PMC1219479

[18] Kong KF Yokosuka T Canonigo-Balancio AJ Isakov N Saito T Altman A. A motif in the V3 domain of the kinase PKC-θ determines its localization in the immunological synapse and functions in T cells via association with CD28. Nat Immunol (2011) 12(11):1105–12. 10.1038/ni.2120 21964608 PMC3197934

[19] Steinberg SF. Structural basis of protein kinase C isoform function. Physiol Rev (2008) 88(4):1341–78. 10.1152/physrev.00034.2007 18923184 PMC2899688

[20] Xu ZB Chaudhary D Olland S Wolfrom S Czerwinski R Malakian K Catalytic domain crystal structure of protein kinase C-θ (PKCθ). J Biol Chem (2004) 279(48):50401–9. 10.1074/jbc.M409216200 15364937

[21] Seco J Ferrer-Costa C Campanera JM Soliva R Barril X. Allosteric regulation of PKCθ: understanding multistep phosphorylation and priming by ligands in AGC kinases. Proteins (2012) 80(1):269–80. 10.1002/prot.23205 22072623

[22] Chuang HC Lan JL Chen DY Yang CY Chen YM Li JP The kinase GLK controls autoimmunity and NF-κB signaling by activating the kinase PKC-[theta] in T cells. Nat Immunol (2011) 12(11):1113–8. 10.1038/ni.2121 21983831

[23] Liu Y Graham C Li A Fisher RJ Shaw S. Phosphorylation of the protein kinase C-theta activation loop and hydrophobic motif regulates its kinase activity, but only activation loop phosphorylation is critical to in vivo nuclear-factor-kappaB induction. Biochem J (2002) 361(Pt 2):255–65. 10.1042/bj3610255 11772397 PMC1222305

[24] Wang X Chuang HC Li JP Tan TH. Regulation of PKC-θ function by phosphorylation in T cell receptor signaling. Front Immunol (2012) 3:197. 10.3389/fimmu.2012.00197 22798961 PMC3393885

[25] Oancea E Meyer T. Protein kinase C as a molecular machine for decoding calcium and diacylglycerol signals. Cell (1998) 95(3):307–18. 10.1016/S0092-8674(00)81763-8 9814702

[26] Meller N Elitzur Y Isakov N. Protein kinase C-θ (PKCθ) distribution analysis in hematopoietic cells: proliferating T cells exhibit high proportions of PKCθ in the particulate fraction. Cell Immunol (1999) 193(2):185–93. 10.1006/cimm.1999.1478 10222061

[27] Pfeifhofer-Obermair C Thuille N Baier G. Involvement of distinct PKC gene products in T cell functions. Front Immunol (2012) 3:220. 10.3389/fimmu.2012.00220 22888329 PMC3412260

[28] Monks CR Kupfer H Tamir I Barlow A Kupfer A. Selective modulation of protein kinase C-θ during T-cell activation. Nature (1997) 385(6611):83–6. 10.1038/385083a0 8985252

[29] Fu G Gascoigne NR. The role of protein kinase Cη in T cell biology. Front Immunol (2012) 3:177. 10.3389/fimmu.2012.00177 PMC3384082 22754555

[30] Monks CR Freiberg BA Kupfer H Sciaky N Kupfer A Three-dimensional segregation of supramolecular activation clusters in T cells. Nature (1998) 395(6697):82–6. 10.1038/25764 9738502

[31] Freiberg BA Kupfer H Maslanik W Delli J Kappler J Zaller DM Staging and resetting T cell activation in SMACs. Nat Immunol (2002) 3(10):911–7. 10.1038/ni836 12244310

[32] Kong KF Altman A. In and out of the bull’s eye: protein kinase Cs in the immunological synapse. Trends Immunol (2013) 34(5):234–42. 10.1016/j.it.2013.01.002 23428395 PMC3647024

[33] Hofinger E Sticht H Multiple modes of interaction between Lck and CD28. J Immunol (2005) 174(7):3839–40. 10.4049/jimmunol.174.7.3839-a 15778335

[34] Thuille N Heit I Fresser F Krumböck N Bauer B Leuthaeusser S Critical role of novel Thr-219 autophosphorylation for the cellular function of PKCθ in T lymphocytes. EMBO J (2005) 24(22):3869–80. 10.1038/sj.emboj.7600856 16252004 PMC1283955

[35] Dustin ML. T-cell activation through immunological synapses and kinapses. Immunol Rev (2008) 221(1):77–89. 10.1111/j.1600-065X.2008.00589.x 18275476

[36] Sims TN Soos TJ Xenias HS Dubin-Thaler B Hofman JM Waite JC Opposing effects of PKCθ and WASp on symmetry breaking and relocation of the immunological synapse. Cell (2007) 129(4):773–85. 10.1016/j.cell.2007.03.037 17512410

[37] Sun Z Arendt CW Ellmeier W Schaeffer EM Jean Sunshine M Gandhi L PKC-θ is required for TCR-induced NF-κB activation in mature but not immature T lymphocytes. Nature (2000) 404(6776):402–7. 10.1038/35006090 10746729

[38] Lin X O’Mahony A Mu Y Geleziunas R Greene WC. Protein kinase C-θ participates in NF-κB activation induced by CD3-CD28 costimulation through selective activation of IκB kinase β. Mol Cell Biol (2000) 20(8):2933–40. 10.1128/MCB.20.8.2933-2940.2000 10733597 PMC85537

[39] Coudronniere N Villalba M Englund N Altman A. NF-κB activation induced by T cell receptor/CD28 costimulation is mediated by protein kinase C-θ. Proc Natl Acad Sci U S A (2000) 97(7):3394–9. 10.1073/pnas.97.7.3394 10716728 PMC16250

[40] Baier-Bitterlich G Uberall F Bauer B Fresser F Wachter H Grunicke H Protein kinase C-theta isoenzyme selective stimulation of the transcription factor complex AP-1 in T lymphocytes. Mol Cell Biol (1996) 16(4):1842–50. 8657160 10.1128/mcb.16.4.1842 PMC231171

[41] Pfeifhofer C Kofler K Gruber T Tabrizi NG Lutz C Maly K Protein kinase C θ affects Ca2+ mobilization and NFAT activation in primary mouse T cells. J Exp Med (2003) 197(11):1525–35. 10.1084/jem.20020234 12782715 PMC2193906

[42] So T Croft M. Regulation of the PKCθ-NF-κB axis in T lymphocytes by the tumor necrosis factor receptor family member OX40. Front Immunol (2012) 3:133. 10.3389/fimmu.2012.00133 22654884 PMC3361009

[43] Sutcliffe EL Li J Zafar A Hardy K Ghildyal R McCuaig R Chromatinized protein kinase C-θ: can it escape the clutches of NF-κB? Front Immunol (2012) 3:260. 10.3389/fimmu.2012.00260 22969762 PMC3428636

[44] Marsland BJ Soos TJ Späth G Littman DR Kopf M. Protein Kinase C θ Is critical for the development of in vivo T helper (Th)2 cell but not Th1 cell responses. J Exp Med (2004) 200(2):181–9. 10.1084/jem.20032229 15263025 PMC2212016

[45] Hayashi K Altman A. Protein kinase C theta (PKCθ): a key player in T cell life and death. Pharmacol Res (2007) 55(6):537–44. 10.1016/j.phrs.2007.04.009 17544292 PMC2045646

[46] Berg-Brown NN Gronski MA Jones RG Elford AR Deenick EK Odermatt B PKCθ signals activation versus tolerance in vivo. J Exp Med (2004) 199(6):743–52. 10.1084/jem.20031022 15024044 PMC2212730

[47] Giannoni F Lyon AB Wareing MD Dias PB Sarawar SR. Protein kinase C θ is not essential for T-cell-mediated clearance of murine gammaherpesvirus 68. J Virol (2005) 79(11):6808–13. 10.1128/JVI.79.11.6808-6813.2005 15890920 PMC1112139

[48] Stevens L Htut TM White D Li X Hanidu A Stearns C Involvement of GATA3 in protein kinase C θ-induced Th2 cytokine expression. Eur J Immunol (2006) 36(12):3305–14. 10.1002/eji.200636400 17111354

[49] Valenzuela JO Iclozan C Hossain MS Prlic M Hopewell E Bronk CC PKCθ is required for alloreactivity and GVHD but not for immune responses toward leukemia and infection in mice. J Clin Invest (2009) 119(12):3774–86. 10.1172/JCI39692 19907075 PMC2786796

[50] Ohkura N Kitagawa Y Sakaguchi S. Development and maintenance of regulatory T cells. Immunity (2013) 38(3):414–23. 10.1016/j.immuni.2013.03.002 23521883

[51] Welters MJP Kenter GG de Vos van Steenwijk PJ Löwik MJG Berends-van der Meer DMA Essahsah F Success or failure of vaccination for HPV16-positive vulvar lesions correlates with kinetics and phenotype of induced T-cell responses. Proc Natl Acad Sci U S A (2010) 107(26):11895–9. 10.1073/pnas.1006500107 20547850 PMC2900675

[52] Seddiki N Brezar V Draenert R. Cell exhaustion in HIV-1 infection: role of suppressor cells. Curr Opin HIV AIDS (2014) 9(5):452–8. 10.1097/COH.0000000000000087 25010895

[53] Sakaguchi S Miyara M Costantino CM Hafler DA. FOXP3+ regulatory T cells in the human immune system. Nat Rev Immunol (2010) 10(7):490–500. 10.1038/nri2785 20559327

[54] Furtado GC Curotto de Lafaille MA Kutchukhidze N Lafaille JJ. Interleukin 2 signaling is required for CD4(+) regulatory T cell function. J Exp Med (2002) 196(6):851–7. 10.1084/jem.20020190 12235217 PMC2194060

[55] Delpoux A Yakonowsky P Durand A Charvet C Valente M Pommier A TCR signaling events are required for maintaining CD4 regulatory T cell numbers and suppressive capacities in the periphery. J Immunol (2014) 193(12):5914–23. 10.4049/jimmunol.1400477 25381435

[56] König S Probst-Kepper M Reinl T Jeron A Huehn J Schraven B First insight into the kinome of human regulatory T cells. PLoS One (2012) 7(7):e40896. 10.1371/journal.pone.0040896 22815858 PMC3397934

[57] Gupta S Manicassamy S Vasu C Kumar A Shang W Sun Z. Differential requirement of PKC-θ in the development and function of natural regulatory T cells. Mol Immunol (2008) 46(2):213–24. 10.1016/j.molimm.2008.08.275 18842300 PMC2700121

[58] Roybal KT Wülfing C. Inhibiting the inhibitor of the inhibitor: blocking PKC-θ to enhance regulatory T cell function. Sci Signal (2010) 3(132):e24–24. 10.1126/scisignal.3132pe24 20664063

[59] Ma J Ding Y Fang X Wang R Sun Z. Protein kinase C-θ inhibits inducible regulatory T cell differentiation via an AKT-Foxo1/3a–dependent pathway. J Immunol (2012) 188(11):5337–47. 10.4049/jimmunol.1102979 22539794 PMC3484896

[60] Kong KF Fu G Zhang Y Yokosuka T Casas J Canonigo-Balancio AJ Protein kinase C-η controls CTLA-4-mediated regulatory T cell function. Nat Immunol (2014) 15(5):465–72. 10.1038/ni.2866 24705298 PMC4040250

[61] Salek-Ardakani S So T Halteman BS Altman A Croft M. Protein kinase Ctheta controls Th1 cells in experimental autoimmune encephalomyelitis. J Immunol (2005) 175(11):7635–41. 10.4049/jimmunol.175.11.7635 16301673

[62] Anderson K Fitzgerald M Dupont M Wang T Paz N Dorsch M Mice deficient in PKC theta demonstrate impaired in vivo T cell activation and protection from T cell-mediated inflammatory diseases. Autoimmunity (2006) 39(6):469–78. 10.1080/08916930600907954 17060026

[63] Tan SL Zhao J Bi C Cynthia Chen X Hepburn DL Wang J Resistance to experimental autoimmune encephalomyelitis and impaired IL-17 production in protein kinase C theta-deficient mice. J Immunol (2006) 176(5):2872–9. 10.4049/jimmunol.176.5.2872 16493044

[64] Healy AM Izmailova E Fitzgerald M Walker R Hattersley M Silva M PKC-theta-deficient mice are protected from Th1-dependent antigen-induced arthritis. J Immunol (2006) 177(3):1886–93. 10.4049/jimmunol.177.3.1886 16849501

[65] Marsland BJ Nembrini C Grün K Reissmann R Kurrer M Leipner C TLR ligands act directly upon T cells to restore proliferation in the absence of protein kinase C-theta signaling and promote autoimmune myocarditis. J Immunol (2007) 178(6):3466–73. 10.4049/jimmunol.178.6.3466 17339441

[66] Cooper JD Smyth DJ Smiles AM Plagnol V Walker NM Allen JE Meta-analysis of genome-wide association study data identifies additional type 1 diabetes risk loci. Nat Genet (2008) 40(12):1399–401. 10.1038/ng.249 18978792 PMC2635556

[67] Raychaudhuri S Remmers EF Lee AT Hackett R Guiducci C Burtt NP Common variants at CD40 and other loci confer risk of rheumatoid arthritis. Nat Genet (2008) 40(10):1216–23. 10.1038/ng.233 18794853 PMC2757650

[68] Stahl EA Raychaudhuri S Remmers EF Xie G Eyre S Thomson BP Genome-wide association study meta-analysis identifies seven new rheumatoid arthritis risk loci. Nat Genet (2010) 42(6):508–14. 10.1038/ng.582 20453842 PMC4243840

[69] Zhernakova A Stahl EA Trynka G Raychaudhuri S Festen EA Franke L Meta-analysis of genome-wide association studies in celiac disease and rheumatoid arthritis identifies fourteen non-HLA shared loci. PLoS Genet (2011) 7(2):e1002004. 10.1371/journal.pgen.1002004 21383967 PMC3044685

[70] Valencia X Stephens G Goldbach-Mansky R Wilson M Shevach EM Lipsky PE. TNF downmodulates the function of human CD4+CD25hi T-regulatory cells. Blood (2006) 108(1):253–61. 10.1182/blood-2005-11-4567 16537805 PMC1895836

[71] Roychoudhuri R Eil RL Restifo NP. The interplay of effector and regulatory T cells in cancer. Curr Opin Immunol (2015) 33C:101–11. 10.1016/j.coi.2015.02.003 25728990

[72] Blay P Astudillo A Buesa JM Campo E Abad M García-García J Protein kinase C theta is highly expressed in gastrointestinal stromal tumors but not in other mesenchymal neoplasias. Clin Cancer Res (2004) 10(12 Pt 1):4089–95. 10.1158/1078-0432.CCR-04-0630 15217944

[73] Duensing A Joseph NE Medeiros F Smith F Hornick JL Heinrich MC Protein kinase C theta (PKCtheta) expression and constitutive activation in gastrointestinal stromal tumors (GISTs). Cancer Res (2004) 64(15):5127–31. 10.1158/0008-5472.CAN-04-0559 15289315

[74] Debiec-Rychter M Wasag B Stul M De Wever I Van Oosterom A Hagemeijer A Gastrointestinal stromal tumours (GISTs) negative for KIT (CD117 antigen) immunoreactivity. J Pathol (2004) 202(4):430–8. 10.1002/path.1546 15095270

[75] Kang GH Kim KM Park CK Kang DY PKC-theta expression in Ewing sarcoma/primitive neuroectodermal tumour and malignant peripheral nerve sheath tumour. Histopathology (2009) 55(3):368–9. 10.1111/j.1365-2559.2009.03368.x 19723158

[76] Ali AS Ali S El-Rayes BF Philip PA Sarkar FH. Exploitation of protein kinase C: a useful target for cancer therapy. Cancer Treat Rev (2009) 35(1):1–8. 10.1016/j.ctrv.2008.07.006 18778896

[77] Abbas W Herbein G. T-cell signaling in HIV-1 infection. Open Virol J (2013) 7:57–71. 10.2174/1874357920130621001 23986795 PMC3751038

[78] Smith BL Krushelnycky BW Mochly-Rosen D Berg P. The HIV nef protein associates with protein kinase C theta. J Biol Chem (1996) 271(28):16753–7. 10.1074/jbc.271.17.9906 8663223

[79] López-Huertas MR Mateos E íaz-Gil GD ómez-Esquer FG Sánchez del Cojo M Alcamí J Protein kinase Cθ is a specific target for inhibition of the HIV type 1 replication in CD4+ T lymphocytes. J Biol Chem (2011) 286(31):27363–77. 10.1074/jbc.M110.210443 21669868 PMC3149330

[80] Bermejo M López-Huertas MR Hedgpeth J Mateos E Rodríguez-Mora S Maleno MJ Analysis of protein kinase C theta inhibitors for the control of HIV-1 replication in human CD4+ T cells reveals an effect on retrotranscription in addition to viral transcription. Biochem Pharmacol (2015) 94(4):241–56. 10.1016/j.bcp.2015.02.009 25732195

[81] Marsland BJ Nembrini C Schmitz N Abel B Krautwald S Bachmann MF Innate signals compensate for the absence of PKC-θ during in vivo CD8+ T cell effector and memory responses. Proc Natl Acad Sci U S A (2005) 102(40):14374–9. 10.1073/pnas.0506250102 16186501 PMC1242314

[82] Budde K Sommerer C Becker T Asderakis A Pietruck F Grinyo JM Sotrastaurin, a novel small molecule inhibiting protein kinase C: first clinical results in renal-transplant recipients. Am J Transplant (2010) 10(3):571–81. 10.1111/j.1600-6143.2009.02980.x 20121745

[83] Friman S Arns W Nashan B Vincenti F Banas B Budde K Sotrastaurin, a novel small molecule inhibiting protein-kinase C: randomized phase II study in renal transplant recipients. Am J Transplant (2011) 11(7):1444–55. 10.1111/j.1600-6143.2011.03538.x 21564523

[84] Skvara H Dawid M Kleyn E Wolff B Meingassner JG Knight H The PKC inhibitor AEB071 may be a therapeutic option for psoriasis. J Clin Invest (2008) 118(9):3151–9. 10.1172/JCI35636 18688284 PMC2496962

[85] Matz M Weber U Mashreghi MF Lorkowski C Ladhoff J Kramer S Effects of the new immunosuppressive agent AEB071 on human immune cells. Nephrol Dial Transplant (2010) 25(7):2159–67. 10.1093/ndt/gfp775 20100729

[86] Bigaud M Wieczorek G Beerli C Audet M Blancher A Heusser C Sotrastaurin (AEB071) alone and in combination with cyclosporine a prolongs survival times of non-human primate recipients of life-supporting kidney allografts. Transplantation (2012) 93(2):156–64. 10.1097/TP.0b013e31823cf92f 22179400

[87] Kamo N Shen XD Ke B Busuttil RW Kupiec-Weglinski JW. Sotrastaurin, a protein kinase C inhibitor, ameliorates ischemia and reperfusion injury in rat orthotopic liver transplantation. Am J Transplant (2011) 11(11):2499–507. 10.1111/j.1600-6143.2011.03700.x 21883905 PMC3625141

[88] Evenou JP Wagner J Zenke G Brinkmann V Wagner K Kovarik J The potent protein kinase C-selective inhibitor AEB071 (sotrastaurin) represents a new class of immunosuppressive agents affecting early T-cell activation. J Pharmacol Exp Ther (2009) 330(3):792–801. 10.1124/jpet.109.153205 19491325

[89] Haarberg KM Li J Heinrichs J Wang D Liu C Bronk CC Pharmacologic inhibition of PKCα and PKCθ prevents GVHD while preserving GVL activity in mice. Blood (2013) 122(14):2500–11. 10.1182/blood-2012-12-471938 23908466 PMC3790515

[90] Graff JR McNulty AM Hanna KR Konicek BW Lynch RL Bailey SN The protein kinase Cβ–selective inhibitor, enzastaurin (LY317615.HCl), suppresses signaling through the AKT pathway, induces apoptosis, and suppresses growth of human colon cancer and glioblastoma xenografts. Cancer Res (2005) 65(16):7462–9. 10.1158/0008-5472.CAN-05-0071 16103100

[91] Jourdan E Leblond V Maisonneuve H Benhadji KA Hossain AM Nguyen TS A multicenter phase II study of single-agent enzastaurin in previously treated multiple myeloma. Leuk Lymphoma (2013) 55(9):2013–7. 10.3109/10428194.2013.861066 24180331

[92] Robertson MJ Kahl BS Vose JM de Vos S Laughlin M Flynn PJ Phase II study of enzastaurin, a protein kinase C beta inhibitor, in patients with relapsed or refractory diffuse large B-cell lymphoma. J Clin Oncol (2007) 25(13):1741–6. 10.1200/JCO.2006.09.3146 17389337

[93] Bronk CC Yu XZ Beg AA. Targeting PKCθ in alloreactivity and graft-versus-host-disease: unanswered questions and therapeutic potential. Front Immunol (2012) 3:259. 10.3389/fimmu.2012.00259 22912640 PMC3418525

[94] Clark RD. Through the looking glass: adventures in kinase inhibitor design and optimization. J Med Chem (2013) 56(5):1796–8. 10.1021/jm400243u 23442161

[95] Jimenez JM Boyall D Brenchley G Collier PN Davis CJ Fraysse D Design and optimization of selective protein kinase C θ (PKCθ) inhibitors for the treatment of autoimmune diseases. J Med Chem (2013) 56(5):1799–810. 10.1021/jm301465a 23398373

[96] Akber U Na BR Ko YS Lee HS Kim HR Kwon MS Phytocomponent 4-hydroxy-3-methoxycinnamaldehyde ablates T-cell activation by targeting protein kinase C-θ and its downstream pathways. Int Immunopharmacol (2015) 25(1):130–40. 10.1016/j.intimp.2015.01.020 25637768

